# N-Acetylcysteine Restores Sevoflurane Postconditioning Cardioprotection against Myocardial Ischemia-Reperfusion Injury in Diabetic Rats

**DOI:** 10.1155/2016/9213034

**Published:** 2015-12-13

**Authors:** Jiefu Lin, Tingting Wang, Yalan Li, Mengxia Wang, Haobo Li, Michael G. Irwin, Zhengyuan Xia

**Affiliations:** ^1^Department of Anesthesiology, The First Affiliated Hospital of Jinan University, Guangzhou 510642, China; ^2^Department of Anesthesiology, The University of Hong Kong, Pokfulam, Hong Kong; ^3^Department of Anesthesiology, Union Hospital, Tongji Medical College, Huazhong University of Science and Technology, Wuhan 430022, China; ^4^Department of Anesthesiology, Affiliated Hospital of Guangdong Medical College, Zhanjiang 524023, China

## Abstract

The effect of sevoflurane postconditioning (sevo-postC) cardioprotection is compromised in diabetes which is associated with increased oxidative stress. We hypothesized that antioxidant N-Acetylcysteine may enhance or restore sevo-postC cardioprotection in diabetes. Control or streptozotocin-induced Type 1 diabetic rats were either untreated or treated with N-Acetylcysteine for four weeks starting at five weeks after streptozotocin injection and were subjected to myocardial ischemia-reperfusion injury (IRI), in the absence or presence of sevo-postC. Diabetes showed reduction of cardiac STAT3 activation (p-STAT3) and adiponectin with concomitantly increase of FoxO1 and CD36, which associated with reduced sevo-postC cardioprotection. N-Acetylcysteine and sevo-postC synergistically reduced the infarct size in diabetic groups. N-Acetylcysteine remarkably increased cardiac p-STAT3 which was further enhanced by sevo-postC. N-Acetylcysteine but not sevo-postC decreased myocardial FoxO1 while sevo-postC but not N-Acetylcysteine significantly increased myocardiac adiponectin in diabetic rats. It is concluded that late stage diabetic rats displayed reduction of cardiac p-STAT3, adiponectin deficiency, and increase of FoxO1 and CD36 expression, which may be responsible for the loss of myocardial responsiveness to sevo-postC cardioprotection. N-Acetylcysteine restored Sevo-postC cardioprotection in diabetes possibly through enhancing cardiac p-STAT3 and adiponectin and reducing Fox1 and CD36.

## 1. Introduction

Ischemic heart disease (IHD) is the main cause of morbidity and mortality in diabetes. Patients with diabetes are particularly at risk of perioperative myocardial infarction with consequent ischemia and reperfusion injury (IRI). Sevoflurane postconditioning (sevo-postC) has been shown to attenuate myocardial IRI by restraining the adhesion of inflammatory cells, blocking up free oxygen radicals, and preventing calcium overload [1]. However, the beneficial effect of sevo-PostC is markedly attenuated by diabetes and the underlying mechanism is unclear. Diabetes can cause impairments to both phosphatidylinositol 3-kinase- (PI3K-) Akt and Janus kinase (Jak-) STAT3, which are the two classic signaling pathways of myocardial protection mechanisms and thus obstruct the postconditioning of myocardium [2, 3]. Recent studies indicated that the inability of insulin to restore sevo-postC cardioprotection in diabetes might be attributed to diabetes-induced STAT3 mediated inhibition of PI3K signaling [4]. Interestingly, our previous study has demonstrated that antioxidant N-Acetylcysteine (NAC) can partially restore the activation of Akt and STAT3 and subsequently attenuated myocardial IRI in the early stage of diabetic rats [5, 6]. However, whether NAC can restore the cardioprotection of sevo-IPostC through the restoration of STAT3 mediated signaling remains unknown.

FoxO transcription factors are important targets of insulin in the myocardium [7, 8]. Three subfamily members (FoxO1, O3, and O4) are critical for cardiac function [8] and regulation of metabolism [9]. FoxO1 is dominant in the adult heart. Cardiac specific activation of FoxO1 adversely alters cardiac metabolism [10]. Studies have shown that cardiac FoxO1 is increased in diabetes [11]. Deletion of FoxO1 in cardiomyocytes shifts their metabolic substrate usage from fatty acid (FA) to glucose and improves cardiac function in subjects with insulin resistance [10]. In addition, Akt phosphorylates/inactivates FoxO and confers cellular protection [12]. Inactivation of FoxO1 also needs STAT3 [13]. These investigations suggest that FoxO1 is the downstream target of both Akt and STAT3 and may play a critical role in determining the progressive increase in myocardial susceptibility to IRI in diabetic hearts.

Oxidative stress level is related to the expression of adiponectin (APN), one of adipocytokines which protect myocardium from IR [14]. Abundant evidence shows that decreased APN is closely correlated with insulin resistance, diabetes, and heart diseases. Our studies have shown that NAC partially attenuated diabetic myocardial IRI through restoration of APN mediated Akt and STAT3 activation [6]. CD36 is known as one of scavenger receptors B and serves as a critical factor in APN metabolism. It is mainly expressed in macrophagocytes and plays an important role in lipid metabolism [15–17]. Studies showed that CD36 could promote FA transfer under pathologic status [18, 19]. Hyperglycemia leads to a dose-dependent upregulation of CD36 [20, 21], which was associated with the uptake of FA in myocardium and contributed to cardiac contractile dysfunction [22]. In addition, studies have shown that APN negatively regulated FoxO1 [23]. FoxO increases mitochondrial CD36 [24, 25]. However, the roles of APN, FoxO1, and CD36 in sevo-postC with or without NAC pretreatment in diabetes have not been studied.

Therefore, we implement NAC treatment to 8-week diabetic rats during feeding period and sevo-postC before reperfusion to test the hypothesis that NAC and sevo-postC can synergistically reduce myocardial IRI in diabetes and explore the roles of p-STAT3, FoxO1, APN, and CD36 in this procedure.

## 2. Methods

The experiment was permitted by the Moral and Ethical Committee of Jinan University. Laboratory animals were raised and used observing the Regulations for the Administration of Affairs concerning Experimental Animals of Guangdong Province.

### 2.1. Establishment of T1DM Model

Male Sprague-Dawley rats (300–350 g) were obtained from the Medical Laboratory Animal Center of Guangdong and fed adaptively for 1 week. T1DM was induced by a single intraperitoneal injection of streptozotocin (Sigma, USA) at the dose of 65 mg/kg. Blood glucose was tested by a One Touch Ultra Vue glucometer (Johnson, USA) 72 hours after the injection. Rats with a blood glucose level higher than 16.7 mmol/L were deemed T1DM.

### 2.2. Experimental Protocols

All rats were randomly divided into 7 groups as follows:

  Sham: non-diabetic control rats implemented thoracotomy without IR.
  I (control): non-diabetic control rats implemented IR.
  I + S: non-diabetic control rats subjected to IR and sevo-postC.
  T + I: T1DM rats implemented IR.
  T + I + S: T1DM rats subjected to IR and sevo-postC.
  T + I + N: T1DM rats treated with NAC and subjected to IR.
  T + I + S + N: T1DM rats treated with NAC and subjected to IR and sevo-postC.

NAC (1.5 g/kg/day) was dissolved in drinking water [5] from 5 to 8 weeks after T1DM establishment. Sevo-postC was carried out by persistently inhalation of 2% sevoflurane for 15 min before reperfusion. Food and water intake were record daily. Weight and blood glucose were measured weekly. Ischemia and reperfusion duration were, respectively, set as 30 min and 90 min. Blood samples were collected from the inferior vena cava at the end of reperfusion and plasma was extracted and stored at −80°C before detection. The ventricular tissue was immediately frozen in liquid nitrogen and conserved at −80°C until assayed.

### 2.3. Myocardial Ischemia-Reperfusion In Vivo and Sevoflurane Postconditioning

Rats were anaesthetized by intraperitoneal injection of pentobarbital (50 mg/kg) and implemented tracheal intubation. Mechanical ventilation was given using a ALC-V9B animal ventilator (Alcott Biotech, China). Ischemia was achieved by ligation of the exposed left anterior descending coronary artery (LAD) for 30 min after thoracotomy. Sevo-postC was accomplished by persistent inhalation of 2% sevoflurane (Abbott, USA) through the tracheal cannula for 15 min in the latter half of ischemia, followed by 90 min of reperfusion.

### 2.4. Determination of Infarct Size

Rats' hearts were dyed by jugular vein injection of Evans Blue (Amresco, USA) at the end of reperfusion and then cut off and stored at −80°C. The hearts were sliced and immersed in 1% 2,3,5-triphenyltetrazolium chloride (TTC, Sigma, USA) solution for 30 min, making the area at risk (AAR) stained brick-red and the normal area stained blue. Infarcted myocardium which located in the AAR remained unstained. Infarct size (IS) was signified as a percentage of AAR [26].

### 2.5. Detection of Plasma Troponin I (TnI) and Creatine Kinase-MB (CK-MB)

Plasma TnI value was estimated by an Ultra Troponin I determination kit (Siemens, USA). Plasma CK-MB levels were detected by a rat CK-MB elisa kit (Roche, Swedish).

### 2.6. Western Blotting

Rat hearts were assembled from all groups followed by the separation of heart tissues of the AAR. Heart tissues were homogenized using lysis buffer, sonicated, and centrifuged at 12 000 g for 20 min at 4°C. Protein concentrations were determined using the Bradford assay (Bio-Rad, USA) and processed for western blotting analysis as described [27, 28]. The primary antibodies against STAT3, p-STAT3 (Y705), FoxO1, CD36 (Abcam, England), APN (CST, American), and GAPDH (Beyotime, China) were used to detect corresponding protein expression of the myocardium. Protein bands were observed by an Immobilon Western Chemiluminescent HRP Substrate (Millipore, USA) and then processed by gray scanning using Image J (National Institutes of Health, USA).

### 2.7. Statistical Analysis

Experimental data was conveyed in the shape of mean ± SEM. Differences between groups and within group were analyzed with one-way ANOVA test followed by LSD test for pairwise comparisons of means using SPSS 13.0 (SPSS, USA). *P* < 0.05 was considered significant.

## 3. Results

### 3.1. General Parameters

As shown in Table 1, diabetic rats displayed increased food intake, water intake, and blood glucose and decreased body weight, compared with control group (all *P* < 0.01). NAC treatment markedly reduced water intake in diabetic rats (*P* < 0.01, T + I + N or T + I + S + N versus T + I). Compared with T + I group, T + I + S + N group showed decreased food intake and water intake without significant impact on body weight during 5~8 weeks of diabetes. NAC had no effect on blood glucose levels in diabetic rats when compared with T + I group.

### 3.2. Infarct Size

**Figure 1:**
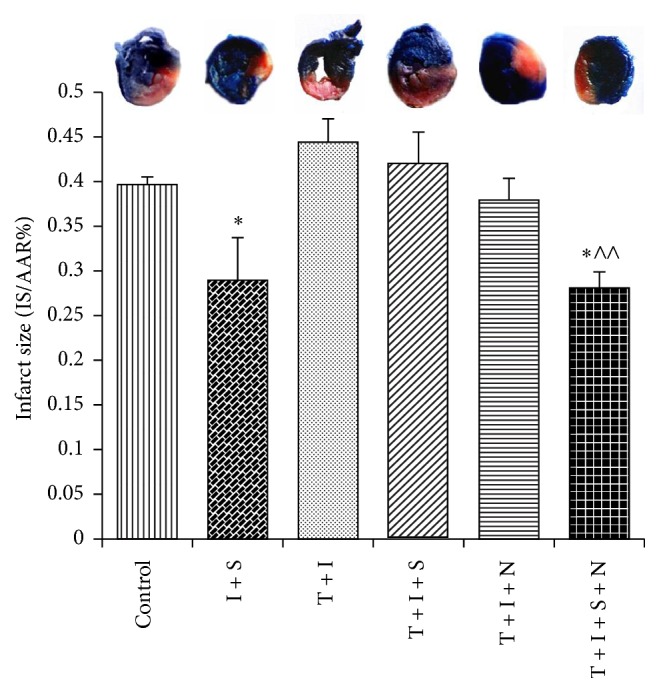
Comparison of IS/AAR% of rat hearts after 30 min ischemia followed by 90 min reperfusion. Data was expressed as mean ± SEM (*n* = 6 per group). I, T, S, and N, respectively, stand for ischemia-reperfusion (I), Type 1 diabetes (T), sevoflurane (S), and N-Acetylcysteine (N); control: ischemia-reperfusion untreated group. ^*∗*^ *P* < 0.05 compared with control group; ^∧∧^ *P* < 0.01 compared with T + I group.

Measurements of infarct size after myocardial IRI were displayed in Figure 1. Compared to control, both I + S and T + I + S + N groups showed decreased infarct size compared with control group (*P* < 0.05). NAC treatment together with sevo-postC dramatically decreased the infarct size of diabetic rats, as compared with the T + I group (*P* < 0.01). NAC or sevo-postC alone did not have the noticeable influence on the IS/AAR of diabetic hearts when compared with T + I group.

### 3.3. Plasma CK-MB and cTnI Levels

**Figure 2:**
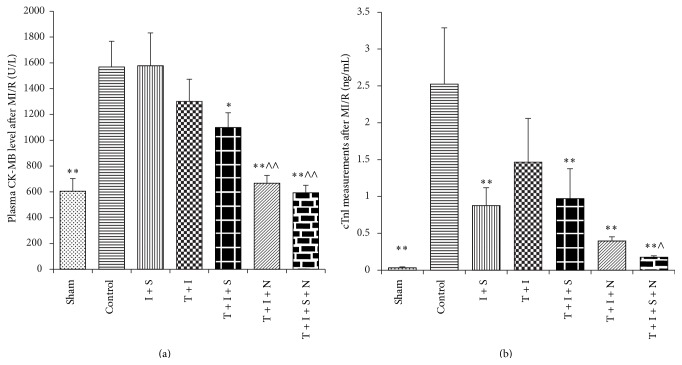
Plasma CK-MB (a) and cTnI (b) measurements after MIR. Plasma was, respectively, separated from blood samples immediately collected after reperfusion. Data was expressed as mean ± SEM (*n* = 12 per group), I, T, S, and N, respectively, stand for ischemia-reperfusion (I), Type 1 diabetes (T), sevoflurane (S), and N-Acetylcysteine (N); control: ischemia-reperfusion untreated group. ^*∗*^ *P* < 0.05; ^*∗∗*^ *P* < 0.01 compared with control group; ^∧^ *P* < 0.05 and ^∧∧^ *P* < 0.01 compared with T + I group.

As indicated in Figure 2(a), ischemia followed by reperfusion significantly increased plasma CK-MB level and cTnI level in control group (*P* < 0.05, control versus Sham). Sevo-postC exerted no effect on CK-MB levels, while NAC largely reduced CK-MB release in diabetic rats with or without sevo-postC (*P* < 0.05, T + I + N or T + I + S + N versus T + I). As shown in Figure 2(b), sevo-postC significantly decreased cTnI levels in control group (*P* < 0.01, I + S versus control). Sevo-postC or NAC alone moderately decreased cTnI levels in diabetic rats, but the differences did not reach statistical significance (*P* > 0.05, T + I + S or T + I + N versus T + I). The synergy of NAC and sevo-postC significantly reduced CK-MB and cTnI levels in diabetic groups (*P* < 0.01, T + I + S + N versus T + I).

### 3.4. The Protein Expression of p-STAT3, FoxO1, APN, and CD36

**Figure 3:**
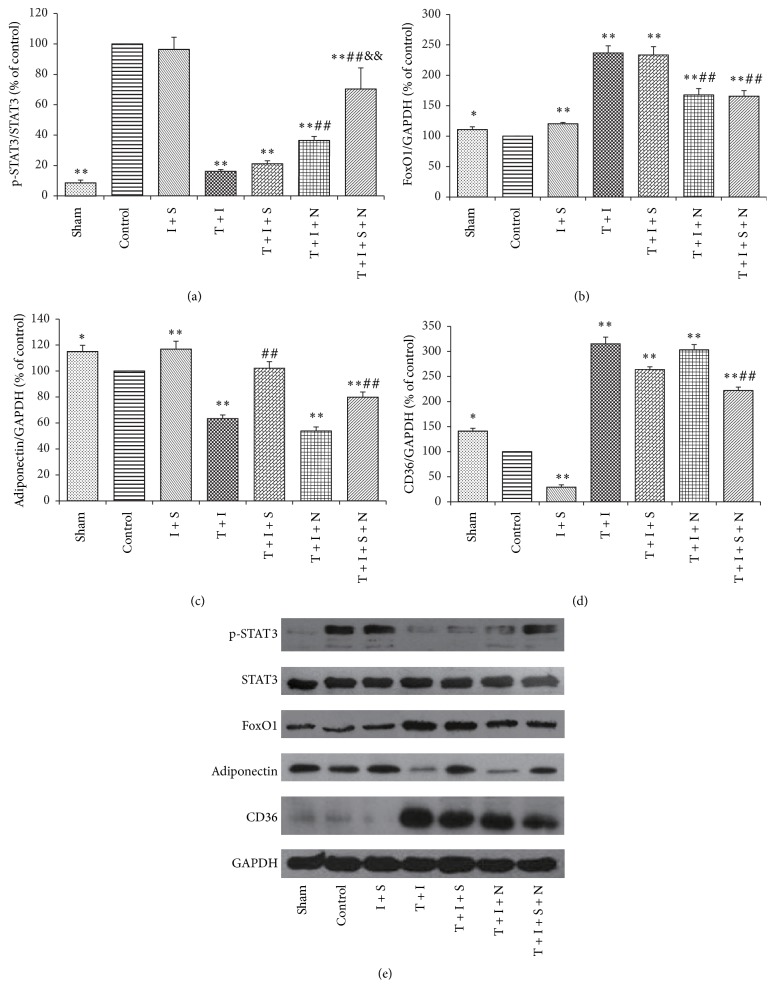
Expression of myocardial p-STAT3 (a), FoxO1 (b), adiponectin (c), CD36 (d), and pictorial diagram of western blots (e) after myocardial IR. Heart tissues were stored at −80°C right after reperfusion. Data was expressed as mean ± SEM (*n* = 6 per group). I, T, S, and N, respectively, stand for ischemia-reperfusion (I), Type 1 diabetes (T), sevoflurane (S), and N-Acetylcysteine (N); control: ischemia-reperfusion untreated group. ^*∗*^ *P* < 0.05; ^*∗∗*^ *P* < 0.01 compared with control group; ^##^ *P* < 0.01 compared with T + I group. ^&&^ *P* < 0.01 compared with T + I + N group.

As shown in Figure 3(a), p-STAT3 displayed lower expression in all groups except I + S when compared with control group (*P* < 0.01 versus control). Diabetes showed significantly decreased p-STAT3 when compared with control group. Sevo-postC made no difference in p-STAT3 either in diabetic rat hearts or in normal ones. NAC remarkably increased STAT3 phosphorylation (*P* < 0.01, T + I + N versus T + I) and the combination of NAC and sevo-postC further increased STAT3 phosphorylation in diabetic myocardium (*P* < 0.01, T + I + N + S versus T + I or versus T + I + N). As described in Figure 3(b), all of the diabetic groups showed increased Fox1 expression when compared with control group (*P* < 0.05). NAC had a prominent effect on the decrease of myocardial FoxO1 expression in diabetic rats with or without sevo-postC (*P* < 0.01, T + I + N or T + I + S + N versus T + I). In Figure 3(c), APN appeared of obvious higher level in normal rats with sevo-postC than in the control rats (*P* < 0.01). Sevo-postC significantly increased myocardiac APN content in diabetic rats with or without NAC (*P* < 0.01, T + I + S or T + I + S + N versus T + I). According to Figure 3(d), CD36 expressions were extremely increased in diabetic groups compared to control group (*P* < 0.01). Sevo-postC markedly reduced CD36 level in normal rat myocardium (*P* < 0.01, I + S versus control), while the combination of NAC and sevo-postC significantly reduced myocardial CD36 expression in diabetic rats (*P* < 0.01, T + I + S + N versus T + I).

## 4. Discussion

Numerous clinical treatments including thrombolytic therapy, coronary artery bypass surgery, and cardiopulmonary resuscitation can cause myocardial IRI. As an ideal inhaled anesthetic, sevoflurane has been widely studied and proved to have a protective effect on myocardium by means of preconditioning or postconditioning implemented on laboratory animals [1, 29]. Resistance induced by sevo-postC against myocardial IRI in diabetic hearts remains to be confirmed, and its mechanism is still not well explored. In the present study, we found that, in the late stage (8 weeks of diabetes) of STZ-induced diabetes, rats displayed the inactivation of STAT3, APN deficiency, and the increase of FoxO1 and CD36 expression. Interestingly, the cardioprotective effect of sevo-postC was lost in diabetic rats, which was restored by the combination of NAC treatment. The possible mechanism may be related to the increased expression of p-STAT3, APN restoration, and the decreased expression of Fox1 and CD36 in diabetic rats.

Excessive oxidative stress, which can be accentuated by hyperglycemia in diabetes, has been declared to aggravate myocardial injury after IR [30]. Since NAC can reduce myocardial dysfunction in diabetic heart [31], two groups of the diabetic rats in the current study were treated with NAC during the later stage (5 to 8 weeks) of the disease before being subjected to IR with or without sevo-postC. In this study, we have observed that sevo-postC can decrease the myocardial IRI in the normal rats reflexed as the decrease of infarct size and cTnI release. In the severe or late stage of diabetic rats, synergy between NAC and sevo-postC was found to protect the myocardium against IRI reflexed as the decrease of infarct size and CK-MB and cTnI secretion, while NAC or sevo-IPostC alone did not help resist myocardial IRI among diabetic groups. Interestingly, inconsistent with infarct size and cTnI results, plasma CK-MB levels showed no significant difference between control and I + S groups. CK-MB had been regarded as a diagnostic criterion of myocardial infarction after acute myocardial infarction (AMI) [32, 33]. However, CK-MB release of patients who suffered AMI did not reach a peak instantly but about 10 hours after ischemia, and this peak value could be used to predict the myocardial infarction which maximized 5~7 days after reperfusion [32]. A similar conclusion was that, in diabetic rats, plasma CK-MB and expansion of myocardial infarct size did not achieve their highest levels simultaneously [34]. These investigations suggested that myocardial infarct size could not always be estimated precisely by CK-MB since CK-MB release can be influenced by traumas such as tracheotomy, venipuncture, and thoracotomy [35]. Since cTnI values and the infarct size were defined as the golden standards evaluating the severity of ischemic injury, our results indeed confirmed the synergistically cardioprotection of NAC and sevo-postC in diabetic rats.

Many studies have demonstrated that sevoflurane can cause the cerebrovascular carbon dioxide reactivity and delayed recovery of vecuronium neuromuscular block in patients with diabetes [36, 37]. However, there are few reports regarding cardioprotection induced by sevoflurane under the condition of diabetes. Currently, the molecular mechanism about resistance effects of sevo-postC against IRI is mainly focused on PI3K-Akt, ERK1/2, and GSK-3*β*, while Jak-STAT3 signaling pathway, which plays essential roles in myocardial protection of postconditioning cardioprotection, is not well investigated. As a downstream target of PI3K/Akt, FoxO1 can presumably increase the sensibility of IRI in diabetic heart. In this study, sevo-postC made no influence on p-STAT3, while myocardial FoxO1 expression was enhanced in normal rats after myocardial IR. In diabetes, the activation of myocardial STAT3 was markedly decreased, whereas the expression of FoxO1 was upregulated. The combination of NAC and sevo-postC significantly increased STAT3 phosphorylation and reduced FoxO1 levels in diabetic rat hearts, revealing a protective effect against myocardial IRI. Studies have shown that increased FoxO1 plays important roles in antioxidation mediated by Sirt1 [38] and reduced myocardial IRI in mice without diabetes [39]. The cardioprotection induced by sphingosine-1-phosphate vanished that was accompanied with the sustaining activation of FoxO1 in STAT-3 deficient mice [13]. In the present study, cardiac FoxO1 was suppressed accompanied with intensive STAT3 phosphorylation in diabetic rats when administrated with NAC and sevo-IPostC, which suggested that the possible mechanism may be associated with STAT3 mediated FoxO1 signaling pathway. However, the effects of FoxO1 and/or STAT3/FoxO1 signaling on myocardial IRI under normal or diabetic state require more research to confirm.

APN is a protein that sensitizes insulin signaling and improves metabolism, protecting against cardiac IRI [40, 41]. However, increasing APN is also related to exacerbating heart failure both in nondiabetic and in diabetic patients [42]. FoxO1, whose activation can be negatively regulated by APN [23], is an important mediator of diabetic cardiac dysfunction [10]. FoxOs also increase mitochondrial CD36, which is the predominant cardiac FA transporter and enhances FA uptake [25]. In our study, we found that cardiac FoxO1 and CD36 are overexpressed with reduction of APN in diabetic rats. The high expressions of FoxO1 and CD36 in diabetic groups displayed an aggravating cardiac injury and suggested the correlation between FoxO1, CD36, and lipid metabolism disorder in diabetes. The combination of NAC and sevo-postC significantly decreased cardiac FoxO1 and CD36 expression and restored APN expression, which contributed to the attenuation of myocardial IRI. These findings indicated that APN, FoxO1, and CD36 may functionally interact in NAC and sevo-postC mediated cardioprotection in diabetes, but their potential interplay in affecting diabetic myocardial metabolism and sensitivity to IRI and IPostC needs further study.

## 5. Conclusions

In summary, this study demonstrated that, in the late stage of STZ-induced diabetes, rats displayed the inactivation of STAT3, APN deficiency, and the increase of FoxO1 and CD36 expression. Cardioprotection induced by sevo-postC was abolished in diabetic rats. NAC and sevo-postC confer synergy in reducing myocardial IRI in diabetes and the possible mechanism may be associated with the increased expression of p-STAT3, APN restoration, and the decreased expression of Fox1 and CD36 in diabetic rats. The proposed studies will help facilitate the development of novel and better therapies for the management of ischemic heart disease in diabetics in whom this disease is very common. It should be noted that the occurrence of ischemia-reperfusion injury is a complex pathological process that involves inflammation and oxidative stress, and the potential attenuation of postischemic inflammation and/or enhancement of endogenous antioxidant enzymes might be attributable to the synergy cardioprotection of join NAC and sevo-postC treatment that deserves further studies.

## Figures and Tables

**Table 1:** General parameters during feeding period.

Groups	Food intake (g/kg/day)		Water intake (mL/kg/day)		Weight (g)		Blood glucose (mmol/L)	
	1–4 weeks	5–8 weeks	1–4 weeks	5–8 weeks	1–4 weeks	5–8 weeks	1–4 weeks	5–8 weeks
Sham	73 ± 4.9	69.1 ± 3.5	149.6 ± 5.4	165.6 ± 9.7	361.3 ± 13.0	433.0 ± 18.0	5.5 ± 0.3	5.3 ± 0.4
Control	76.5 ± 3.5	70.1 ± 1.5	154.5 ± 2.1	156.3 ± 0.6	360.5 ± 11.6	432.0 ± 11.2	5.4 ± 0.4	5.9 ± 0.3
I + S	67.2 ± 4.6	66.6 ± 2.2	155.2 ± 3.2	150.3 ± 3.0	370.0 ± 13.7	274.3 ± 9.2^*∗∗*^	5.5 ± 0.3	5.7 ± 0.2
T + I	177.9 ± 24.1^*∗∗*^	187.3 ± 2.9^*∗∗*^	887.9 ± 158.3^*∗∗*^	1118.8 ± 12.2^*∗∗*^	271.3 ± 10.4^*∗∗*^	238.8 ± 0.9^*∗∗*^	28.0 ± 2.0^*∗∗*^	29.5 ± 1.1^*∗∗*^
T + I + S	180.8 ± 19.4^*∗∗*^	206.3 ± 1.6^*∗∗*^	927.8 ± 170.5^*∗∗*^	1224.0 ± 10.5^*∗∗*^	274.3 ± 9.2^*∗∗*^	271.3 ± 10.4^*∗∗*^	26.9 ± 2.1^*∗∗*^	26.1 ± 1.5^*∗∗*^
T + I + N	188.8 ± 24.2^*∗∗*^	170.6 ± 6.5^*∗∗*^	1072.5 ± 78.2^*∗∗*^	614.1 ± 130.8^*∗∗*&&^	254.8 ± 15.7^*∗∗*^	253.5 ± 4.6^*∗∗*^	29.1 ± 1.2^*∗∗*^	27.4 ± 2.5^*∗∗*^
T + I + N + S	192.5 ± 8.0^*∗∗*^	154.5 ± 16.5^*∗∗*&^	1110.9 ± 52.5^*∗∗*^	677.0 ± 105.2^*∗∗*&&^	253.3 ± 10.3^*∗∗*^	269.5 ± 13.8^*∗∗*^	26.3 ± 2.0^*∗∗*^	27.5 ± 1.7^*∗∗*^

Data was expressed as mean ± SEM (*n* = 12); I, T, S, and N, respectively, stand for ischemia-reperfusion (I), Type 1 diabetes (T), sevoflurane (S), and N-Acetylcysteine (N); control: ischemia-reperfusion untreated group.

^*∗∗*^ *P* < 0.01 compared with control group; ^&^ *P* < 0.05 and ^&&^ *P* < 0.01 compared with T + I group.
